# Selected nasogastric lavage in patients with nonvariceal upper gastrointestinal bleeding

**DOI:** 10.1186/s12876-021-01690-z

**Published:** 2021-03-06

**Authors:** Eun Jeong Gong, Li-chang Hsing, Hyun Il Seo, Myeongsook Seo, Baek Gyu Jun, Jong Kyu Park, Sang Jin Lee, Koon Hee Han, Young Don Kim, Woo Jin Jeong, Gab Jin Cheon, Min-Ju Kim

**Affiliations:** 1 Department of Internal Medicine, Gangneung Asan Hospital, University of Ulsan College of Medicine, Gangneung, Korea; 2 Department of Gastroenterology, Asan Medical Center, Seoul, Korea; 3 Department of Clinical Epidemiology and Biostatistics, Asan Medical Center, Seoul, Korea; 4 Department of Internal Medicine, Gangneung Asan Hospital, University of Ulsan College of Medicine, 38 Bangdong-gil, Sacheon-Myeon, Gangwon-do 25440 Gangneung, Korea

**Keywords:** Endoscopic hemostasis, Gastric lavage, Peptic ulcer hemorrhage, Treatment outcome

## Abstract

**Background:**

Risk stratification before endoscopy is crucial for proper management of patients suspected as having upper gastrointestinal bleeding (UGIB). There is no consensus regarding the role of nasogastric lavage for risk stratification. In this study, we investigated the usefulness of nasogastric lavage to identify patients with UGIB requiring endoscopic examination.

**Methods:**

From January 2017 to December 2018, patients who visited the emergency department with a clinical suspicion of UGIB and who underwent nasogastric lavage before endoscopy were eligible. Patients with esophagogastric variceal bleeding were excluded. The added predictive ability of nasogastric lavage to the Glasgow–Blatchford score (GBS) was estimated using category-free net reclassification improvement and integrated discrimination improvement.

**Results:**

Data for 487 patients with nonvariceal UGIB were analyzed. The nasogastric aspirate was bloody in 67 patients (13.8 %), coffee-ground in 227 patients (46.6 %), and clear in 193 patients (39.6 %). The gross appearance of the nasogastric aspirate was associated with the presence of UGIB. Model comparisons showed that addition of nasogastric lavage findings to the GBS improved the performance of the model to predict the presence of UGIB. Subgroup analysis showed that nasogastric lavage improved the performance of the prediction model in patients with the GBS ≤ 11, whereas no additive value was found when the GBS was greater than 11.

**Conclusions:**

Nasogastric lavage is useful for predicting the presence of UGIB in a subgroup of patients, while its clinical utility is limited in high-risk patients with a GBS of 12 or more.

**Supplementary Information:**

The online version contains supplementary material available at 10.1186/s12876-021-01690-z.

## Background

Nonvariceal upper gastrointestinal bleeding (UGIB) is currently the main cause of hospital admission and often requires emergency endoscopy [1]. As gastrointestinal (GI) bleeding is associated with significant morbidity and mortality, accurate assessment and timely management, including endoscopic hemostasis, are important to reduce further bleeding and to improve clinical outcomes [2, 3]. Although endoscopic procedures are considered to be safe, concerns have been raised regarding emergency endoscopy because it is usually performed during non-regular working hours with limited resources and assistance. In line with this, the importance of identifying patients at risk for UGIB requiring early endoscopy is increasingly being recognized in the initial management of patients suspected of having UGIB.

Several factors such as presenting with symptoms of hematemesis or melena, a bloody nasogastric aspirate, and laboratory data are known to be useful indicators for UGIB [4]. In addition, various risk stratification schemes, including the Glasgow–Blatchford score (GBS), Rockall score, and AIMS65, have been developed and validated to identify high-risk patients requiring emergency endoscopy [5–9]. Nasogastric lavage is an intuitive and simple bedside maneuver that provides information regarding the probability of UGIB, with a high positive predictive value [10, 11]. However, its negative predictive value is low, and the insertion of a nasogastric tube can cause considerable discomfort to the patients [12–14].

Currently, there is no consensus regarding the role of nasogastric lavage for risk stratification of patients with UGIB before endoscopy [15–17]. A few studies have evaluated the clinical utility of nasogastric lavage in patients with UGIB, showing that a bloody nasogastric aspirate was associated with the presence of active bleeding [8–11, 18–20]. However, uncertainty remains about its exact role in clinical practice, as most studies have suggested an association between the gross appearance of the nasogastric aspirate and the clinical diagnosis but could not clarify who needs nasogastric lavage and who does not. In this study, we aimed to investigate the usefulness of nasogastric lavage in patients presumed to have nonvariceal UGIB and to determine which patients could benefit from nasogastric lavage.

## Methods

From January 2017 to December 2018, consecutive adult patients who visited the emergency department with a clinical suspicion of UGIB and underwent endoscopic examination were eligible. Patients with a history of hematemesis, melena, hematochezia, dizziness, or a combination of these symptoms were included. Patients who were younger than 18 years, those who initially visited another hospital and were referred to our center for further management of a confirmed bleeding episode, or those with esophagogastric variceal bleeding were excluded. After excluding 153 patients who did not undergo nasogastric lavage before endoscopy, 487 patients were included in the analysis (Additional file 1: Fig. 1).

The demographic and historical data, physical examination findings, laboratory data, endoscopic findings, and outcome data were collected from the medical records. The results of nasogastric lavage were categorized as clear, coffee-ground, or bloody, according to the gross appearance of the nasogastric aspirate, and a coffee-ground or bloody appearance was considered as a positive finding.

The GBS was calculated to stratify patients with UGIB. The GBS is based on the systolic blood pressure, heart rate, hemoglobin and blood urea nitrogen levels at presentation; the presence of melena or syncope; and the presence or absence of hepatic disease or cardiac failure. The total score ranges from 0 to 23, with higher scores indicating a higher risk of further bleeding or death [5, 21]. For subgroup analysis, patients were divided into three groups according to the GBS, so that the number of patients in each group was similar: (1) GBS ≤ 7 (n = 167), (2) 7 < GBS ≤ 11 (n = 153), and (3) GBS > 11 (n = 167). In addition, the ratio of blood urea nitrogen to creatinine (BUN/Cr ratio) was calculated [4]. The final diagnosis was based on endoscopic findings, and the source of UGIB was determined. The possible bleeding focus included all lesions with active bleeding or stigmata of recent bleeding, such as peptic ulcers, Dieulafoy’s lesion, Mallory-Weiss tear, malignancy, acute gastric mucosal lesion, and angioectasia. All endoscopic procedures were performed by a fellow or board-certified endoscopist and were supported by nurses with skills in assisting with therapeutic endoscopy.

Patients received intravenous proton pump inhibitor (PPI) therapy (pantoprazole or esomeprazole) with an initial bolus injection of 80 mg, followed by an 8 mg/hour infusion prior to endoscopy. Intravenous PPI infusion was discontinued when the endoscopic examination revealed a non-ulcer etiology or when there was no evidence of GI bleeding. Endoscopic hemostasis was performed using various techniques, including thermal coagulation and mechanical therapy, with or without injection therapy. The mode of therapy was chosen at the discretion of the endoscopists. Uncontrolled bleeding despite endoscopic hemostasis was considered an indication for angiographic embolization or surgery. The study protocol was approved by the Institutional Review Board of the Gangneung Asan Hospital (number 2020-03-009).

### Statistical analysis

Continuous variables are shown as median (range), and categorical variables are shown as number (percentage). Differences in baseline characteristics were tested by the chi-square test, Fisher’s exact test, t-test, or Mann–Whitney *U-*test, as appropriate. A logistic regression model was used to identify individual correlates associated with the presence of a bleeding focus, and odds ratio (OR) and 95 % confidence interval (CI) were estimated. To investigate the added predictive ability of a new variable to the GBS, category-free net reclassification improvement (cNRI) and integrated discrimination improvement (IDI) were calculated [22]. In addition, the receiver operative characteristic (ROC) curve and the area under the ROC curve (AUROC) were estimated. All statistical analyses were performed by using SPSS v21.0 (SPSS Inc., Chicago, USA) and R version 3.6.1 (http://www.r-project.org), and a two-sided *p* value less than 0.05 was considered statistically significant. R packages of the ‘PredictABEL’ and ‘pROC’ were used in this study [23].

## Results

### Study population

The baseline demographic and clinical characteristics of the study population are summarized in Table 1 and Additional file 3: Table 1. The median age was 69 years (range 18–94 years), and 53.4 % were male. More than three-quarters of the patients had comorbidities, including coronary artery disease, chronic kidney disease, and liver cirrhosis. In addition, 29.6 % of the patients were taking antithrombotic agents or anticoagulants at the time of admission. Regarding the presenting symptoms, 88.9 % of the patients complained of melena or hematemesis. Hypotension (systolic blood pressure < 100 mmHg) and tachycardia (heart rate > 100 per minute) were found in 23.6 % and 43.9 % of patients, respectively. The BUN/Cr ratio was greater than 30 in 52.0 % of patients, and the median GBS was 10 (range 0–19).

**Table 1:** Baseline characteristics of the study population (N = 487)

Age, median (range), years	69 (18–94)
Male	260 (53.4)
Comorbidities	374 (76.8)
Diabetes mellitus	120 (24.6)
Cerebrovascular accident	57 (11.7)
Coronary artery disease	50 (10.3)
Chronic kidney disease	27 (5.5)
Liver cirrhosis	56 (11.5)
Previous peptic ulcer disease	63 (12.9)
Antithrombotic/anticoagulant use	144 (29.6)
Aspirin	101 (20.7)
Clopidogrel	36 (7.4)
Dual antiplatelet therapy	20 (4.1)
Warfarin	9 (1.8)
DOAC	20 (4.1)
NSAIDs use	43 (8.8)
Steroid use	10 (2.1)

*DOAC* direct oral anticoagulant, NSAIDs, non-steroidal anti-inflammatory drugs

## Nasogastric aspirate and endoscopic findings

The gross appearance of the nasogastric aspirate before endoscopy was bloody in 67 patients (13.8 %), coffee-ground in 227 patients (46.6 %), and clear in 193 patients (39.6 %) (Additional file 3: Table 1). The median time from admission to endoscopy was 3.3 hours (range 0.4–55.9 h), and endoscopy was performed within 24 hours of presentation in 95.9 % of cases. On endoscopy, 229 patients (47.0 %) had peptic ulcer disease, 41 patients (8.4 %) had Mallory-Weiss syndrome, and 35 patients (7.2 %) had malignancy (Table 2). Other diagnoses included marginal ulcer, esophageal ulcer, angioectasia, gastrointestinal stromal tumor, acute gastric mucosal lesion, and duodenal tuberculosis. On the other hand, no evident bleeding focus was found in 151 patients (31.0 %). Age, comorbidity, and antithrombotic agent or anticoagulant use did not differ between patients with and without a bleeding focus.

**Table 2:** Clinical characteristics according to the gross appearance of the nasogastric aspirate

	Total (N = 487)	Nasogastric lavage		*P* value
		Positive (n = 294)^§^	Negative (n = 193)	
Presenting symptoms				< 0.001
Hematemesis	214 (43.9)	175 (59.5)	39 (20.2)	
Melena	219 (45.0)	99 (33.7)	120 (62.2)	
Hematochezia	51 (10.5)	19 (6.5)	32 (16.6)	
Dizziness	3 (0.6)	1 (0.3)	2 (1.0)	
Endoscopic diagnosis				< 0.001
Gastric ulcer	177 (36.3)	122 (41.5)	55 (28.5)	
Duodenal ulcer	52 (10.7)	31 (10.5)	21 (10.9)	
Mallory-Weiss syndrome	41 (8.4)	33 (11.2)	8 (4.1)	
Malignancy	35 (7.2)	23 (7.8)	12 (6.2)	
Others^§§^	31 (6.4)	20 (6.8)	11 (5.7)	
No evidence of bleeding	151 (31.0)	65 (22.1)	86 (44.6)	
Endoscopic hemostasis	150/336 (44.6)	124/229 (54.1)	26/107 (24.3)	< 0.001

^§^Positive includes bloody and coffee-ground nasogastric aspirate

^§§^Others include marginal ulcer, esophageal ulcer, esophagitis, vascular ectasia, gastrointestinal stromal tumor, and tuberculosis

Endoscopic hemostasis was required in 150 of 336 patients (44.6 %), and successful endoscopic hemostasis was possible in 147 patients (98.0 %). A total of 14 patients had GBS < 1, including 10 patients whose bleeding focus was not evident on endoscopy, three patients with Mallory-Weiss syndrome, and one patient with gastric ulcer. Of these, only one patient with Mallory-Weiss syndrome underwent band ligation for the exposed vessel without active bleeding. Otherwise, no endoscopic intervention was required in patients with GBS < 1.

When comparing clinical and laboratory data according to the results of nasogastric lavage, more patients with hematemesis had bloody or coffee-ground nasogastric aspirates compared with those with melena or hematochezia. More patients with a gastric ulcer or Mallory-Weiss syndrome had bloody or coffee-ground nasogastric aspirates, while more patients with duodenal ulcers had clear nasogastric aspirates. The gross appearance of the nasogastric aspirate was significantly associated with the presence of a nonvariceal UGIB (Additional file 2: Fig. 2). In addition, more patients with bloody or coffee-ground nasogastric aspirate required endoscopic hemostasis compared to those with negative findings (54.1 % and 24.3 %, *p* < 0.001).

### Prediction of nonvariceal UGIB using non‐endoscopic variables

**Fig. 1:**
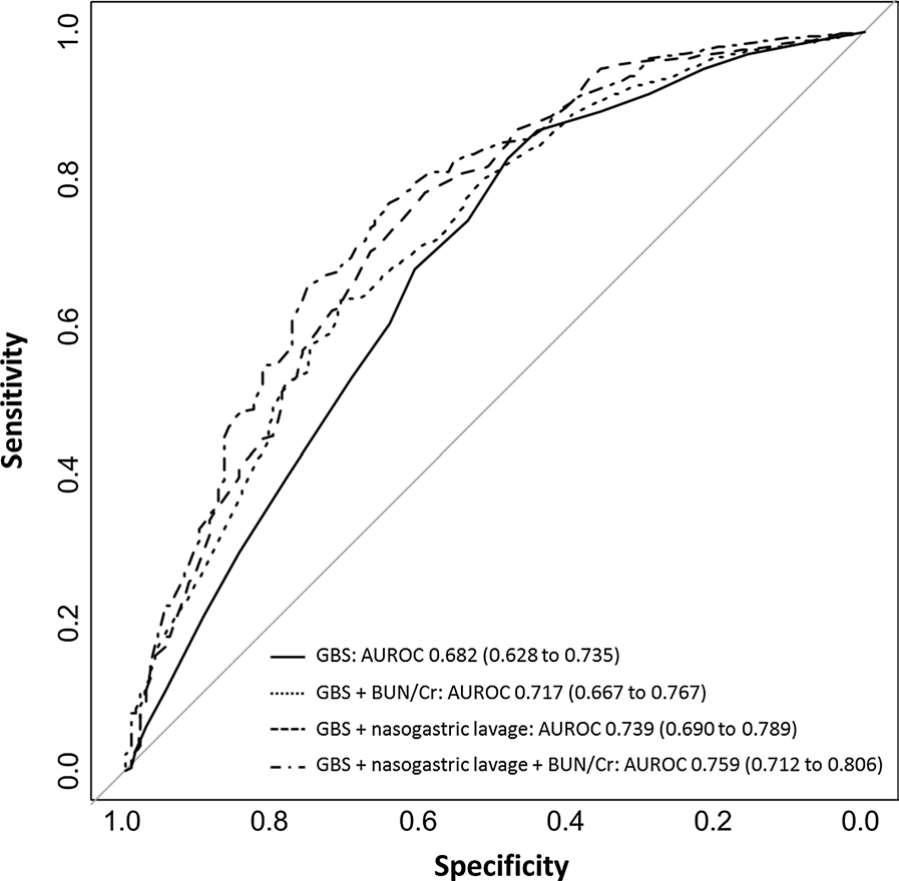
The receiver operating characteristic curves of Glasgow–Blatchford score, BUN/Cr ratio > 30, and nasogastric lavage for predicting the presence of a bleeding focus on endoscopy. AUROC, area under the receiver operating characteristic curve; BUN/Cr, ratio of serum blood urea nitrogen to creatinine > 30 versus ≤ 30; GBS, Glasgow–Blatchford score

Logistic regression analysis showed that the BUN/Cr ratio > 30, a higher GBS score, and a coffee-ground or bloody nasogastric aspirate were factors associated with the presence of nonvariceal UGIB on endoscopy (Additional file 3: Table 2). Comparison of models including the GBS, BUN/Cr ratio, and nasogastric lavage showed that the addition of the BUN/Cr ratio and nasogastric lavage findings was associated with improvement of the performance of the prediction model for the presence of UGIB over GBS alone (Table 3). The combination of the GBS, BUN/Cr ratio, and nasogastric lavage had the cNRI of 0.469 (95 % CI 0.285–0.653), IDI of 0.085 (95 % CI 0.061–0.110), and an AUROC of 0.759 (95 % CI 0.712–0.806) (Fig. 1). Subgroup analysis showed that nasogastric lavage improved the performance of the prediction model in patients with a GBS ≤ 11, whereas it had no additive value when the GBS was greater than 11 (Table 4). The BUN/Cr ratio was associated with added predictive ability in all subgroups regardless of the GBS.

**Table 3:** Model comparisons for the prediction of the presence of a bleeding focus on endoscopy

Variables	cNRI	95 % CI	*P* value	IDI	95 % CI	*P* value
GBS	Reference			Reference		
GBS + BUN/Cr	0.623	0.443–0.803	< 0.001	0.030	0.015–0.045	< 0.001
GBS + NGL	0.502	0.315–0.689	< 0.001	0.060	0.040–0.080	< 0.001
GBS + NGL + BUN/Cr	0.469	0.285–0.653	< 0.001	0.085	0.061–0.110	< 0.001

*BUN/Cr* blood urea nitrogen/creatinine ratio > 30 versus ≤ 30, *CI* confidence interval, *cNRI* category-free net reclassification improvement, *GBS* Glasgow–Blatchford score, *IDI* integrated discrimination improvement, *NGL* nasogastric lavage

**Table 4:** Subgroup analysis of model comparisons for the prediction of the presence of a bleeding focus on endoscopy

Variables	cNRI	95 % CI	*P* value	IDI	95 % CI	*P* value
GBS ≤ 7 (N = 167)						
GBS	Reference			Reference		
GBS + BUN/Cr	0.422	0.158–0.685	0.002	0.027	0.002–0.052	0.032
GBS + NGL	0.602	0.315–0.889	< 0.001	0.101	0.056–0.146	< 0.001
GBS + NGL + BUN/Cr	0.559	0.268–0.850	< 0.001	0.128	0.077–0.180	< 0.001
7 < GBS ≤ 11 (N = 153)						
GBS	Reference			Reference		
GBS + BUN/Cr	0.504	0.149–0.860	0.005	0.043	0.012–0.075	< 0.001
GBS + NGL	0.666	0.329–1.003	< 0.001	0.103	0.065–0.141	< 0.001
GBS + NGL + BUN/Cr	0.676	0.376–0.975	< 0.001	0.134	0.086–0.181	< 0.001
GBS > 11 (N = 167)						
GBS	Reference			Reference		
GBS + BUN/Cr	0.401	0.035–0.766	0.032	0.032	0.002–0.062	0.039
GBS + NGL	0.175	-0.187–0.537	0.343	0.005	-0.007–0.017	0.379
GBS + NGL + BUN/Cr	0.432	0.062–0.801	0.022	0.034	0.003–0.065	0.031

*BUN/Cr* blood urea nitrogen/creatinine ratio > 30 versus ≤ 30, *CI* confidence interval, *cNRI* category-free net reclassification improvement, *GBS* Glasgow–Blatchford score, *IDI* integrated discrimination improvement, *NGL* nasogastric lavage

## Discussion

In this study, we investigated the usefulness of nasogastric lavage for prediction of nonvariceal UGIB and found that the additive value of nasogastric lavage during risk stratification of patients differs by the GBS. The addition of nasogastric lavage findings to the GBS was associated with improved performance of the prediction model over the GBS alone in patients with GBS ≤ 11, whereas nasogastric lavage was not beneficial when applied to patients with GBS ≥ 12. These results suggest that nasogastric lavage is useful and can provide additional information about the possibility of UGIB in a subgroup of patients. By contrast, nasogastric lavage was not helpful in patients suspected to be at high risk of UGIB based on the GBS, and for these patients, endoscopy should not be delayed while nasogastric lavage is performed.

In the emergency room, various factors are used to predict the risk and outcome of UGIB, including symptoms, vital signs, age and comorbidities of the patient, and laboratory data. If emergency endoscopy can be offered 24 hours a day, establishing hemodynamic stability may be all we need before performing endoscopy. However, in some institutions and situations such as night times and weekends, medical resources are limited. Moreover, performing an endoscopy as soon as possible does not always lead to favorable outcomes, and delaying the procedure until the patient is stable is associated with better outcomes than proceeding with endoscopy without resuscitation [24, 25]. Therefore, it is important to identify patients who require urgent endoscopy and those who can benefit from scheduled endoscopy.

Various strategies for risk stratification of patients with GI bleeding have been developed using both endoscopic and non-endoscopic variables [5–7]. In addition, nasogastric lavage is frequently performed in the emergency department to rule in or out GI bleeding [9, 20]. However, the role of nasogastric lavage before endoscopy remains uncertain. As the GBS is a well-established scoring system for predicting the need for intervention in UGIB [26–28], we aimed to estimate the additive value of nasogastric lavage to the GBS, rather than developing an entirely new prediction model.

Several studies have investigated the usefulness of nasogastric lavage in patients with UGIB. In addition to hemodynamic instability and laboratory findings, a bloody nasogastric aspirate was helpful to discriminate patients with UGIB [10, 11, 19]. Others showed that a fresh bloody nasogastric aspirate could be used as a predictor for endoscopic intervention in patients with acute UGIB [8, 9, 18]. However, the insertion of a nasogastric tube is not completely safe and is one of the most painful procedures performed in the emergency department [14, 29]. In addition, since the negative predictive value of nasogastric lavage is low, the routine use of nasogastric lavage prior to endoscopy cannot be recommended. In the present study, we aimed to determine which patients could benefit from nasogastric lavage during risk stratification. As expected, we found that bloody or coffee-ground nasogastric aspirate was associated with the presence of UGIB. A bloody or coffee-ground nasogastric aspirate was a useful predictor for the presence of nonvariceal UGIB when added to the GBS based on the cNRI, IDI, and AUROC. Interestingly, the additive value of nasogastric lavage was not significant in patients with a GBS greater than 11, indicating that the role of nasogastric lavage was limited in a certain subgroup of patients with a high risk of UGIB. This result is similar to that of previous study that the appearance of nasogastric aspirate was most useful in hemodynamically stable patients without hematemesis [9].

The main pitfall of nasogastric lavage is its low negative predictive value. A previous study that analyzed 1498 patients who underwent endoscopy showed that 15.9 % of patients with a clear nasogastric aspirate demonstrated an actively bleeding lesion on endoscopy, and 88.8 % in the clear aspirate group had one or more diagnostic findings [10]. Another study found that one out of 18 patients with a clear nasogastric aspirate had active bleeding on endoscopy, and argued against the usefulness of nasogastric lavage to determine whether there is bleeding and whether endoscopy should be performed [11]. In the present study, 24.3 % of patients with a clear nasogastric aspirate required endoscopic hemostasis, and 44.6 % of patients in this group were found to have the source of bleeding located other than in the duodenum. In clinical practice, physicians may utilize nasogastric lavage to rule out GI bleeding that needs urgent endoscopy. However, a negative result on nasogastric lavage does not guarantee a low possibility of UGIB, and therefore a negative result of nasogastric lavage should be interpreted cautiously during clinical decision making.

Non-endoscopic variables such as the clinical and laboratory parameters are identifiable during the initial evaluation at the emergency department. In a previous study, a ratio of the heart rate to the systolic blood pressure of greater than 1.4 was associated with UGIB [19]. Another study found that hemoglobin level (< 8 g/dL) and white blood cell count (> 12,000/µL) could differentiate patients with UGIB who needed early endoscopy [8]. These factors can be calculated shortly after presentation to hospital and provide information regarding when an endoscopy should be performed and what level of care is appropriate for patients with UGIB. In the present study, a BUN/Cr ratio > 30 was found to be a useful predictor for the presence of UGIB, which is concordant with the findings of previous reports [4, 13]. In addition, this simple and easy to calculate variable had additive value to the GBS in predicting the presence of a bleeding focus, suggesting that there is another variable that can be used to reduce the need for nasogastric lavage in risk stratification of patients with UGIB.

The performance of nasogastric lavage has been reported variously as it has been investigated according to different reference diagnostic criteria; some studies used active bleeding or high-risk stigmata of bleeding as a reference [8, 9, 18], while others considered an adherent clot and other obvious lesions, including erosive gastritis and esophagitis, as the cause of UGIB [10, 11]. In the present study, given that the goal of management of patients with nonvariceal UGIB is not only to deal with active or severe bleeding but to also find any possible cause of bleeding and to prevent recurrent bleeding, we included all lesions that might cause UGIB in the analyses. Despite this, no evident bleeding focus was found on endoscopy in one-third of the study population and 22.1 % of patients with bloody or coffee-ground nasogastric aspirates. These results might reflect real-world practice where it is not unusual to find either multiple possible sources or no definitive source of GI bleeding.

This study has several limitations. Firstly, as a single-center study, selection bias cannot be excluded. Second, since healthcare systems and available resources may differ among countries, our findings may not be generalizable to other clinical settings. Third, patients were divided into three groups according to the number of patients included in the subgroups, and the cutoff value of the GBS could be different in other study populations. Another possible limitation is that patients who did not undergo nasogastric lavage were excluded from the analyses, which may be associated with patient selection bias. However, we do not believe this had a major impact on our study findings, as comparisons between the patients with and without nasogastric lavage showed that most clinical characteristics were similar between the two groups (Additional file 3: Table 3).

## Conclusions

The usefulness of nasogastric lavage is different according to the GBS in patients with nonvariceal UGIB. Nasogastric lavage is useful in predicting the presence of UGIB in a subgroup of patients with GBS ≤ 11, while endoscopic examination without nasogastric lavage is preferred in patients with a GBS greater than 11.

## Supplementary Information

**Additional file 1: Fig. 1**. Flowchart of this study. NVUGIB, non-variceal upper gastrointestinal bleeding

**Additional file 2: Fig. 2**. Association between the gross appearance of the nasogastric aspirate and the presence of a bleeding focus on endoscopy (*p* < 0.001).

**Additional file 3:** **Table 1**. Patient characteristics acording to the presence of a bleeding ocus on endoscopy. **Table 2**. Factors associated with the presence of a bleeding focus on endoscopy. **Table 3**. Baseline characteristics of the patietns visiting the emergency department with complaints suspected of upper gastrointestinal bleeding.

## Data Availability

The datasets generated and/or analyzed during this study are not publicly available given our commitment to patient privacy rights. However, anonymous data may be requested from the corresponding author for valid use.

## Abbreviations

UGIB: upper gastrointestinal bleeding
GI: gastrointestinal
GBS: Glasgow–Blatchford score
BUN: Blood urea nitrogen
Cr: Creatinine
PPI: Proton pump inhibitor
cNRI: Category-free net reclassification improvement
IDI: Integrated discrimination improvement
