# Correction to: Selected nasogastric lavage in patients with nonvariceal upper gastrointestinal bleeding

**DOI:** 10.1186/s12876-021-01860-z

**Published:** 2021-07-12

**Authors:** Eun Jeong Gong, Li-chang Hsing, Hyun Il Seo, Myeongsook Seo, Baek Gyu Jun, Jong Kyu Park, Sang Jin Lee, Koon Hee Han, Young Don Kim, Woo Jin Jeong, Gab Jin Cheon, Min-Ju Kim

**Affiliations:** 1 Department of Internal Medicine, Gangneung Asan Hospital, University of Ulsan College of Medicine, Gangneung, Korea; 2 Department of Gastroenterology, Asan Medical Center, Seoul, Korea; 3 Department of Clinical Epidemiology and Biostatistics, Asan Medical Center, Seoul, Korea; 4 Department of Internal Medicine, Gangneung Asan Hospital, University of Ulsan College of Medicine, 38 Bangdong-gil, Sacheon-Myeon, Gangwon-do 25440 Gangneung, Korea

## Correction to: BMC Gastroenterol (2021) 21:113 https://doi.org/10.1186/s12876-021-01690-z

In the original publication [1] a contribution statement was accidentally removed during the publication process.

This statement is as followed:

Eun Jeong Gong and Li-chang Hsing contributed equally to this work.

The original article has been updated. We apologize to the authors and readers for the inconvenience.
