# Supplementary figures and images for: Selected nasogastric lavage in patients with nonvariceal upper gastrointestinal bleeding
Source: BMC Gastroenterol. 2021 Mar 6;21:113. doi: 10.1186/s12876-021-01690-z (PMC7937281; doi:10.1186/s12876-021-01690-z)

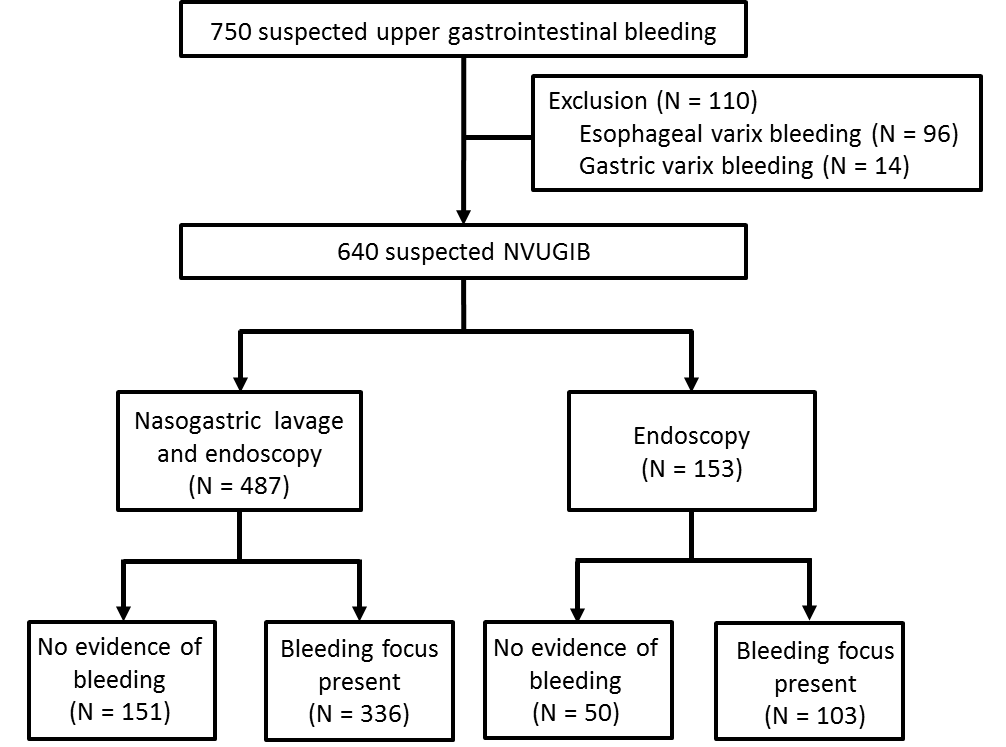

Supplement: Supplementary file 1 — Additional file 1: Fig. 1. Flowchart of this study. NVUGIB, non-variceal upper gastrointestinal bleeding [file 12876_2021_1690_MOESM1_ESM.tif]

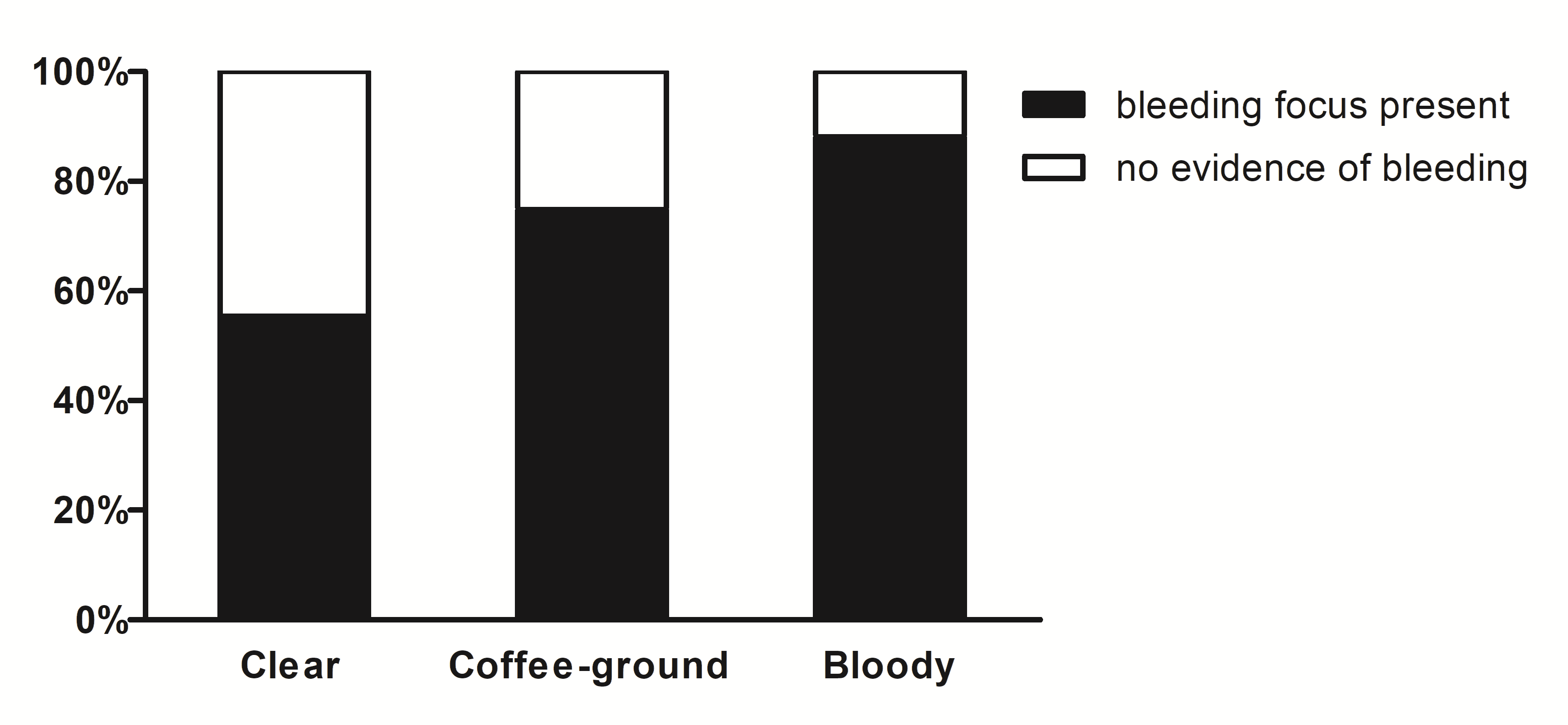

Supplement: Supplementary file 2 — Additional file 2: Fig. 2. Association between the gross appearance of the nasogastric aspirate and the presence of a bleeding focus on endoscopy (p < 0.001). [file 12876_2021_1690_MOESM2_ESM.tif]
