# Supplementary material for: Selected nasogastric lavage in patients with nonvariceal upper gastrointestinal bleeding
Source: BMC Gastroenterol. 2021 Mar 6;21:113. doi: 10.1186/s12876-021-01690-z (PMC7937281; doi:10.1186/s12876-021-01690-z)
Supplement: Supplementary file 3 — Additional file 3: Table 1. Patient characteristics acording to the presence of a bleeding ocus on endoscopy. Table 2. Factors associated with the presence of a bleeding focus on endoscopy. Table 3. Baseline characteristics of the patietns visiting the emergency department with complaints suspected of upper gastrointestinal bleeding. [file 12876_2021_1690_MOESM3_ESM.docx]

**Supplementary Table 1** Patient characteristics according to the presence of a bleeding focus on endoscopy

|  | Total  (N = 487) | Possible source of bleeding | | *P* value |
| --- | --- | --- | --- | --- |
|  |  | Absent  (n = 151) | Present  (n = 336) |  |
| Age, years | 69 (18–94) | 69 (18–92) | 68 (19–94) | 0.696 |
| Presenting symptoms |  |  |  | 0.052 |
| Hematemesis | 214 (43.9) | 62 (41.1) | 152 (45.2) |  |
| Melena | 219 (45.0) | 65 (43.0) | 154 (45.8) |  |
| Hematochezia | 51 (10.5) | 24 (15.9) | 27 (8.0) |  |
| Dizziness | 3 (0.6) | 0 | 3 (0.9) |  |
| SBP <100 mmHg | 115 (23.6) | 22 (14.6) | 93 (27.7) | 0.002 |
| Heart rate >100 beats/min | 214 (43.9) | 52 (34.4) | 162 (48.2) | 0.005 |
| Laboratory findings |  |  |  |  |
| Anemia^§^ | 401 (82.3) | 111 (73.5) | 290 (86.3) | 0.001 |
| BUN/Cr ratio >30 | 253 (52.0) | 46 (30.5) | 207 (61.6) | <0.001 |
| Platelet, ×10^3^/µL | 213 (8–981) | 210 (30–981) | 215 (8–801) | 0.358 |
| Prothrombin time, % | 90 (5–137) | 92 (5–134) | 90 (7–137) | 0.173 |
| Comorbidities | 374 (76.8) | 116 (76.8) | 258 (76.8) | 0.993 |
| Antithrombotic/anticoagulant | 144 (29.6) | 41 (27.2) | 103 (30.7) | 0.433 |
| Glasgow-Blatchford score | 10 (0–19) | 7 (0–19) | 11 (0–19) | <0.001 |
| Nasogastric aspirate |  |  |  | <0.001 |
| Clear | 193 (39.6) | 86 (57.0) | 107 (31.8) |  |
| Coffee-ground | 227 (46.6) | 57 (37.7) | 170 (50.6) |  |
| Bloody | 67 (13.8) | 8 (5.3) | 59 (17.6) |  |

Data present number (percent) or median (range).

BUN/Cr ratio, ratio of blood urea nitrogen to creatinine; SBP, systolic blood pressure

^§^Hemoglobin <13 g/dL for men and <12 g/dL for women**Supplementary Table 2** Factors associated with the presence of a bleeding focus on endoscopy

|  | Univariable analysis | | | Multivariable analysis | | |
| --- | --- | --- | --- | --- | --- | --- |
|  | OR | 95% CI | *P* value | OR | 95% CI | *P* value |
| Age, years | 0.998 | 0.985–1.010 | 0.695 |  |  |  |
| Comorbidities | 0.998 | 0.633–1.573 | 0.993 |  |  |  |
| Antithrombotic agent | 1.186 | 0.774–1.818 | 0.434 |  |  |  |
| BUN/Cr ratio >30 | 3.663 | 2.430–5.521 | <0.001 | 2.429 | 1.535–3.845 | <0.001 |
| Glasgow-Blatchford score | 1.172 | 1.118–1.228 | <0.001 | 1.133 | 1.075–1.193 | <0.001 |
| Nasogastric aspirate |  |  |  |  |  |  |
| Clear | Reference | | | Reference | | |
| Coffee-ground | 2.397 | 1.586–3.623 | <0.001 | 1.975 | 1.266–3.082 | 0.003 |
| Bloody | 5.928 | 2.687–13.075 | <0.001 | 6.731 | 2.944–15.392 | <0.001 |

BUN/Cr ratio, ratio of blood urea nitrogen to creatinine; CI, confidence interval; OR, odds ratio

**Supplementary Table 3** Baseline characteristics of the patients visiting the emergency department with complaints suspected of upper gastrointestinal bleeding

|  | Total  (N = 640) | Nasogastric lavage | | *P* value |
| --- | --- | --- | --- | --- |
|  |  | No (n = 153) | Yes (n = 487) |  |
| Age, median (range), years | 67 (18–101) | 64 (21–101) | 69 (18–94) | 0.008 |
| Male | 352 (55.0) | 92 (60.1) | 260 (53.4) | 0.144 |
| Presenting symptoms |  |  |  | 0.176 |
| Hematemesis | 277 (43.3) | 63 (41.2) | 214 (43.9) |  |
| Melena | 282 (44.1) | 63 (41.2) | 219 (45.0) |  |
| Hematochezia | 77 (12.0) | 26 (17.0) | 51 (10.5) |  |
| SBP <100 mmHg | 143 (22.3) | 28 (18.3) | 115 (23.6) | 0.169 |
| Heart rate >100 beats/min | 277 (43.3) | 63 (41.2) | 214 (43.9) | 0.547 |
| Anemia^§^ | 523 (81.7) | 122 (79.7) | 401 (82.3) | 0.468 |
| BUN/Cr ratio >30 | 316 (49.4) | 60 (41.2) | 253 (52.0) | 0.020 |
| Comorbidities | 483 (75.5) | 109 (71.2) | 374 (76.8) | 0.164 |
| Antithrombotic agent | 174 (27.2) | 30 (19.6) | 144 (29.6) | 0.016 |
| Glasgow-Blatchford score | 10 (0–19) | 10 (0–18) | 10 (0–19) | 0.188 |
| Diagnosis |  |  |  | 0.008 |
| Peptic ulcers | 304 (47.5) | 75 (49.0) | 229 (47.0) |  |
| Mallory-Weiss syndrome | 60 (9.4) | 19 (12.4) | 41 (8.4) |  |
| Malignancy | 40 (6.3) | 5 (3.3) | 35 (7.2) |  |
| Others | 35 (5.5) | 4 (2.6) | 31 (6.4) |  |
| No evidence of bleeding | 201 (31.4) | 50 (32.7) | 151 (31.0) |  |

BUN/Cr ratio, serum blood urea nitrogen/creatinine ratio; SBP, systolic blood pressure.

^§^Hemoglobin <13 g/dL for men and <12 g/dL for women
